# Experiences of healthcare interactions before and after suicidal behaviour among older adults attending geropsychiatric services: an interpretative phenomenological analysis

**DOI:** 10.1136/bmjopen-2025-100636

**Published:** 2025-05-30

**Authors:** Maja Sjöberg, Sara Hed, Stefan Wiktorsson, Anne Ingeborg Berg, Jennifer Strand, Sabrina Doering, Margda Waern

**Affiliations:** 1 University of Gothenburg Institute of Neuroscience and Physiology, Gothenburg, Sweden; 2 Department of Neuropsychiatry, Sahlgrenska University Hospital, Region Västra Götaland, Gothenburg, Sweden; 3 Region Västra Götaland, Department of Psychotic Disorders, Sahlgrenska University Hospital, Gothenburg, Sweden; 4 University of Gothenburg Department of Psychology, Gothenburg, Sweden

**Keywords:** Aged, Suicide & self-harm, Delivery of Health Care, Integrated, QUALITATIVE RESEARCH, Health Services Accessibility

## Abstract

**ABSTRACT:**

**Objectives:**

Physical illness and functional disability are common in older adult populations and strongly linked to suicidal behaviour. The aim was to explore how older adults who engaged in a suicidal act experienced their interactions with healthcare providers.

**Design:**

This study re-examined transcripts from a broader study involving experiences of older adults who took part in two separate semi-structured interviews focusing on their experiences before and after a suicidal act. Interpretative phenomenological analysis was applied.

**Setting:**

A geriatric psychiatric outpatient clinic in a large Swedish city.

**Participants:**

Participants (70+) were recruited among consecutive Swedish-speaking patients in outpatient treatment following a suicidal act within the last 3–36 months. Exclusion criteria were personality disorder, ongoing psychosis, aphasia, delirium, clinical dementia or Montreal Cognitive Assessment score indicating moderate/severe cognitive impairment. Out of 22 eligible, nine accepted participation (four women and five men, age range 71–92 years). Prior to the suicidal act, all had their main care contact in primary care, and all but one were on antidepressants.

**Results:**

Participants described interactions with healthcare services that amplified their feelings of alienation, loneliness, worthlessness and self-stigma. Difficulties accessing care increased their sense of powerlessness. Some participants were cognizant of their mental health needs but experienced obstacles that hindered them from managing their illness, which reduced their sense of agency. These situations increased frustration and hopelessness and contributed to the development of suicidal behaviour. On the contrary, feeling listened to in trustful and validating relationships helped restore self-respect and agency and fostered engagement in their individual suicide preventative strategies.

**Conclusions:**

The findings can inform educational interventions and clinical approaches to the care and management of older adults with symptoms of common mental disorders. Exploring experiences of care interactions before and after suicidal acts across different clinical settings and cultures could be areas for future research.

**Strengths and limitations of this study**

- Our sample size is considered appropriate for the analysis method employed.
- Two separate interviews allowed for reflection and follow-up questions which broadened and deepened the interpretive analysis.
- Relatively many patients declined participation, and experiences may have differed in those who did not wish to take part.
- The results are based on narratives of persons in treatment within the Swedish healthcare system, which impacts transferability to other clinical settings.

## INTRODUCTION

 Older adults have the highest rates of suicide worldwide,^1^ with particularly high rates among men aged 80 and over.^2^,^4^ Older adults represent a quickly growing segment of the population,^5^ making suicide prevention a pressing public health concern.

Physical illness and functional disability are associated with suicidal behaviour in older adults to a larger degree than they are for younger persons,^6 7^ and numerous medical conditions are associated with fatal and non-fatal suicidal behaviours in older adults.^8^ The burden of medical illness and functional issues means that most older adults depend on healthcare providers.^9 10^ However, they may be less likely than younger persons to report depressive symptoms and suicidal behaviour, even when asked specifically about suicidal ideation.^11 12^ Assessment scales used in primary and psychiatric care to screen for depressive symptoms and anxiety are not adapted for older age groups,^13^ meaning that older adults’ mental suffering risks going under the radar. Also, general practitioners (GPs) may not recognise suicidal issues in their older patients because these individuals often present with physical symptoms. As a result, the physician’s attention may be directed towards addressing these complaints, potentially overlooking underlying mental health concerns.^9 14^ When mental ill-health is identified, treatment with psychoactive medication is often prescribed.^15^ Older adults are less likely to have accessed psychiatric care prior to suicide compared with their younger counterparts.^10^ The Swedish National Board of Health and Welfare^15^ has expressed concern that lack of services for older adults with mental health issues may increase mental suffering and ultimately elevate the risk of suicide in this age group.

Applying a psychological autopsy approach, Kjølseth *et al*^16^ interviewed proxy informants (relatives, GPs and home-based care workers) to elucidate how older adults had experienced contact with health services prior to their death by suicide. Respondents reported that older adults who died by suicide expressed distrust in care. Many had experienced dissatisfactory communication; the older adult did not convey their problems, or the healthcare professionals failed to understand them.^16^

To our knowledge, there are only a few interview studies that report subjective experiences of older adults who survived a suicidal act.^17^,^22^ For example, Crocker *et al*^17^ showed that one way in which participants’ experienced lack of control in life (which was regarded as suicidogenic) was by finding their treatments unhelpful. Engaging in a suicidal act was expressed to regain control over their situation. Feeling like a burden to healthcare professionals in the aftermath of a suicidal act was described as a factor that contributed to experiencing life as struggle, which was regarded as a suicidogenic factor.^17^ Wand *et al*^21 22^ explicitly examined older adults’ experiences of interactions with healthcare providers and found that the relational context with their healthcare professionals was relevant both before and after their self-harm. Feeling invalidated and rejected by healthcare professionals could fuel a decision to self-harm and negatively impact the willingness to confide suicidal ideation.^21 22^ A one-sided emphasis on risk management at the expense of listening and person-centred, individualised help following self-harm could increase suffering and even retraumatise individuals who had self-harmed.^20 21^ We recently carried out interviews with older adults with care contact at a geriatric psychiatric service in the aftermath of a suicidal act.^23^ Respondents were eager to talk about how they experienced healthcare. Interactions with health professionals were positive for some, but others described how negative experiences could catalyse a suicidal process. The current study aims to further elaborate on these findings by revisiting the interview transcripts with a specific focus on how the participants perceived and attributed meaning to their interactions with healthcare providers. Specifically, the research question in this study is: how do older adults who have engaged in a suicidal act experience their interactions with healthcare providers before and after the act?

## Methods

The current study is based on interview transcripts from a broader research initiative investigating older adults’ subjective experiences of their suicidal process.^23^ Transcripts were re-examined for this sub-study, now with a specific focus on interactions with healthcare professionals. The study has a qualitative research design and builds on interviews with persons (70+) in treatment within geriatric psychiatric services. The specific questions in the interview guide that are addressed in the current study include: “Did you reach out to anyone prior to the attempt, during or after? What was that like? Was there anything you found helpful? Was there anything you wish had been different? What type of treatment did you receive to help you manage/cope with suicidal thoughts/plans/actions? What has been helpful? What could have been different/more helpful?” Interview content regarding interactions with healthcare professionals that participants brought up at any point in either of the two interviews was also coded.

### Participants and procedure

The study protocol included a baseline assessment carried out by a research psychologist (SW) followed by two semi-structured interviews (1 to 2 weeks apart).^23^ The interviews were conducted by a female psychologist, author SH, and took place in either the participant’s home (eight interviews) or at the outpatient unit (10 interviews).

Participants were recruited among patients in treatment at a geriatric psychiatric outpatient clinic in a large Swedish city in the aftermath of a suicide attempt, as deemed by the assessing psychiatrist and registered as such in the patients’ medical record within the past 3–36 months. It is important to note that for some participants, the psychiatrist’s conclusion regarding suicidal intent differed from the older person’s explanation of the act as described at the time of the qualitative research interview.^23^ Further inclusion criteria were age 70 and above, sufficient communication skills including Swedish language proficiency, being able to provide written informed consent and approval by their psychiatrist that their symptoms were sufficiently stable to take part in the study. Individuals assessed with ongoing high risk of suicide were thus not eligible for inclusion. Persons with personality disorder, ongoing psychosis, aphasia, delirium, a clinical diagnosis of dementia or Montreal Cognitive Assessment (MoCa) score of less than two SD from the Swedish normative score for educational level and age^24^ were excluded. The reason for excluding persons with personality disorders, psychotic illness or dementia was that the interview study was a first step in a larger initiative that would include testing of a brief intervention for suicidal older adults with common mental disorders. Suicidal persons with personality disorders, psychosis or dementia would most likely require more specific interventions. Further, we noted in our previous study of older adults hospitalised after a suicidal act^25^ that cognitively impaired persons experienced some challenges when relating their personal experiences in the research interview.

Mental health professionals at the clinic were informed about the inclusion criteria and aided recruitment by approaching patients they regarded suitable. The potential participants were then approached by a member of the research team, face-to-face or by telephone. 22 persons met the inclusion criteria and 13 declined participation. Reasons for declining included a wish to move on from the suicidal crisis, scarce energy or time limitations due to frequent medical visits and/or hindering life circumstances. Four women and five men (age range 71–91 years, eight born in Sweden) agreed to participate in the study and received oral and written information. All nine completed both interviews.

The first author MS reviewed medical records to complement participants’ narratives with a verifiable account for information regarding psychiatric diagnoses (DSM-5), somatic diagnoses, psychiatric history and healthcare contexts. All fulfilled criteria for at least one psychiatric disorder, and six out of nine had at least one serious physical illness/functional disability, as rated in accordance with the Cumulative Illness Rating Scale for Geriatrics (CIRS-G).^26^ Further, eight out of nine were on antidepressants at the time of the act, and all had such treatment at the time of the interview.

Table 1 shows participant characteristics including psychiatric and medical conditions. For a detailed description of psychopharmacological treatment both at the time of the suicidal act and the time of the interview, see Hed *et al.*^23^ Primary care was the main healthcare setting for all participants at the time of the suicidal act. Some also had contact with specialist physicians. None of the participants had care contacts within psychiatric services at the time of the suicidal act, but five had a previous history of such contact.

**Table 1:** Sociodemographic and clinical characteristics of participants (n=9)

Participants/ pseudonyms	Age group	Months betweenindex SA*and interview	MADRS† score at interview	CIRS-G‡total somatic score at interview	Number of CIRS-G somatic categories with rating3 or 4§ at interview	MoCA¶ score at interview
Martha	< 75	7	0 (no depression)	21	3	25
Kate	≥ 75	4	12 (mild depression)	11	3	24**
Lena	≥ 75	27	22 (moderate depression)	10	2	29
Mary	≥ 75	6	0 (no depression)	10	0	25
Daniel	< 75	14	5 (no depression)	5	0	23**
Robin	≥ 75	16	29 (moderate depression)	11	1	29
Lars	≥ 75	5	27 (moderate depression)	15	3	25
Leo	≥ 75	4	33 (moderate depression)	7	0	24
Hugo	≥ 75	12	4 (no depression)	21	3	28

* SA=Suicidal Act as assessed by attending psychiatrist.

† MADRS=Montgomery-Asberg Depression Rating Scale (0–6=no depression, 7–19=mild depression, 20–34=moderate depression, >34 = severe depression).

‡ CIRS-G=Cumulative Illness Rating Scale for Geriatrics.

§ A rating of 3 or 4 on CIRS-G corresponds to serious physical illness or disability.

¶ MoCA=Montreal Cognitive Assessment.

** Mild Cognitive Impairment.

CIRS-G, Cumulative Illness Rating Scale - Geriatrics ; MADRS, Montgomery-Asberg Depression Rating Scale; MoCA, Montreal Cognitive Assessment; SA, suicidal act.

### Data collection

The baseline assessment included measures of medical illness burden (CIRS-G),^26^ depressive symptoms (MADRS),^27^ suicidal behaviour (Columbia-Suicide Severity Rating Scale)^28^ and cognitive screening (MoCA).^29^

A semi-structured interview guide was developed by the research group, based on previous research on suicidal behaviour in older adults. The interview guide focused on several aspects of the participants’ experience of suicidal behaviour and included specific questions about what kind of treatment and support they had received and their views of such treatment. Having two separate interview occasions allowed the interviewer to review the first transcript and to thereafter formulate follow-up questions for the second interview. The interviews (n=18) lasted 45–90 min. All interviews were audio recorded and transcribed. The transcripts were not returned to participants for comments and/or correction.

### Data analysis

For this study, transcripts were reanalysed and coded with the current research question in focus. Interpretative phenomenological analysis (IPA)^30^ was used to analyse the interviews. IPA was deemed suitable as it aims to obtain an ‘insider’s view’ of the research topic. IPA incorporates ideography, phenomenology and hermeneutics,^30^ which were adhered to during the analytical steps.

MS read, re-read and took phenomenological notes on all but one transcript. The latter was read and coded by the author SH since the author MS had an ongoing treatment relationship with that participant. Notes for each transcript were listed and grouped into preliminary themes, and connections between themes were drawn by the two first authors.

In the second interpretative phase, double hermeneutic was applied. The double hermeneutic involved how we, as researchers, made meaning out of the participants’ meaning making. The interpretative process in IPA also involves the hermeneutic circle, which relies on the assumption that understanding the whole requires an understanding of its individual parts and vice versa. During this phase, phenomenological codes in individual transcripts were interpreted in the context of the whole narrative, and new codes were generated. Five researchers (MS, SH, AIB, SW, MW) who had read and taken notes on the transcripts were involved in this phase.

In the third phase, individual themes were discussed, and a cross-case analysis was conducted. During this process, the research group had numerous discussions to find the most suitable thematic structure. Our ambition with the structure was to find general themes but at the same time adhere to the idiographic stance in IPA. Finally, to ensure that individual cases were interpreted in a way that made sense within the broader context of the interview, SH reviewed the interpretations from her perspective as interviewer. This procedure yielded no changes in the thematic structure. It is important to note that the themes reflect the interplay between each participant’s affects and suicidal feelings and healthcare and that there was no clear time trajectory; the interactions could have taken place both before (sometimes years before) and after the suicidal act. Since interpretation is central to IPA, we acknowledge that there are other potential ways to elucidate meaning from the data. An example of the coding scheme is shown in online supplemental table 1.

### Ethical considerations and reflexivity

This study is a part of a larger research project on older adults’ experiences of their suicidal behaviour, approved by the Gothenburg Regional Ethics Committee (EPN, Dnr 2020–00321). Since it is the same nine participants in the current study as it was in our previous study,^23^ we have changed pseudonyms for the participants to strengthen their anonymity. The first authors (MS and SH) were employed as clinical psychologists at the geropsychiatric clinic where data collection took place and had a special interest and experience of working with older adults in psychiatric care. Neither SH, who carried out the interviews, nor SW, who administered the rating scales, was involved as clinicians for any of the participants in the study. However, participants were informed that the interviewer (SH) worked as a clinical psychologist at the clinic.

The interviewer’s (SH) experience in working with depressed and suicidal older adults was helpful in dealing with participants’ reaction to the sensitive nature of the interviews. Several efforts were made to ensure participant comfort and safety during the data collection process. Even though most participants experienced the interviews in an overall positive manner, some reported a temporal increase in anxiety and mental fatigue. Participants were offered breaks when needed, and the research psychologist paid close attention to their well-being. All participants had a regular care contact whom they could turn to if needed before, during or after the data collection phase. Participants were assured that they could withdraw from the study at any time and that they could take part in the study result after completion.

Since this study uses the same data as the study by Hed *et al*^23^ and is written by the same research group, there was a concern that the interpretation of the transcripts would be influenced by the analysis of the previous study. To counteract this risk, MS did not read the results of the initial publication prior to analysing the transcripts for the current paper. Also, all authors met on numerous occasions to discuss the analysis in relation to the aim of this study and repeatedly returned to the transcripts to make sure themes were grounded on data.

To balance the participants’ meaning making with our interpretations as researchers and clinicians, we initially read the transcripts while attempting to set aside preconceptions. However, in our numerous subsequent discussions, both individual and cross-case interpretations were broadened by our various perspectives and knowledge, in accordance with the IPA method.

### Patient and public involvement

Patients were not involved in the design or conduct of this study. An educator at the non-governmental organisation (NGO) Suicidprevention i Väst contributed during manuscript preparation, with comments regarding both clinical and research implications from an NGO perspective.

## Results

Participants’ experiences of interactions with healthcare in relation to their suicidal episode are summarised in four themes: amplifying vulnerabilities, obstacles cause powerlessness, healthcare that reduces agency and restored hope through trust and validation. The first three themes reflect participants’ dependency on healthcare due to ageing-related health problems and how interactions with healthcare providers could contribute to feelings of sadness, hopelessness, loss of self-worth and, for some, suicidal ideation and behaviour. The last theme, restored hope through trust and validation, reflects how interactions with healthcare providers could potentially be suicide preventative.

### Amplifying vulnerabilities

This theme highlights how participants’ interactions with healthcare professionals increased feelings of burdensomeness and decreased their willingness to seek help. All participants described how their vulnerabilities were met with an unfortunate dynamic that amplified, rather than alleviated, their struggles. The narratives also highlight the influence of stigma and how perceptions of ageing and mental health impact the participants’ help-seeking behaviour and the treatment options they chose in dialogue with healthcare providers. For the participants represented in this study, the reduced trust they felt towards their healthcare providers contributed to unfavourable outcomes.

Mary, for example, had always taken pride in independently fending for herself and dedicating her time to caring for her loved ones. However, she found herself in a situation of dependency, and she felt ashamed and devalued by the doctor.

She [the doctor] was so strict…. So, I left there feeling so sad. I was so sad. Because I wasn’t asking for a miracle—I was just asking for my pills. But she thought the dose was too high, and… she… she kept sitting at the computer the whole time, only occasionally looking at me with this angry expression, and I felt like I was shrinking. (Mary)

Hugo, who suffered from multimorbidity, said that his trust in the healthcare system decreased during the period leading up to his suicidal act. He underwent repeated medical examinations and felt he was being passed around, going in and out of different wards and hospital departments. His experiences with healthcare professionals in specialised medical care often made him feel overlooked and unimportant, which he exemplified with a situation in which the doctor forgot to reinstate his anticoagulant medication following a medical procedure. “They didn’t give me my pills back…so I had two minor strokes”. Despite many setbacks, Hugo continued to strive for improved health. In connection with one of his many hospitalisations, Hugo was discharged from the hospital with no advance notice. The doctor paid no attention to Hugo’s attempts to communicate the severity of his symptoms. Hugo perceived that he had been treated unfairly and was overwhelmed by suicidal thoughts. On return home, he overdosed on medication.

I: If the way they [medical staff] approached you at the time had been different, do you think it would have made a difference?

IF: I wouldn’t have been so disappointed. What affects me the most, I think, and it’s been like this my whole life—is when I’m treated unfairly. That hits me hard. (…) if I’m treated unfairly, I get sad. And I feel like I was treated very unfairly there. (Hugo)

Following their suicidal act, all participants had been referred to geriatric psychiatric care. For some, this was their first experience with psychiatric care facilities, and some expressed feelings of shame associated with being perceived as mentally ill. For some participants, these feelings impacted on care decisions.

I remember one time when you had to sit and wait, you know. Then they brought in someone in handcuffs, escorted by two guards. You know, I thought, damn, that’s how people see me. I remember that—it wasn’t… it wasn’t a great thing to witness. So no, no more ECT [electroconvulsive treatment] now. (Robin)

Living with hypochondria, Leo frequently visited primary care centres, got checked, felt better and went home. His suicide attempt led to an involuntary admission at the psychiatric clinic, an experience that made him feel even more anxious and vulnerable.

I got really scared because I don’t want to be there—I’m not dangerous (…). One time (after discharge), the nice nurse called, and I said that I wasn’t feeling great. So, I wondered, are they going to come and take me now? (Leo)

Other participants expressed complex emotions about being treated in geriatric psychiatric care, where all patients were aged 70 and above. Lars struggled with existential challenges related to ageing, loss of autonomy and vitality and had difficulties identifying himself as an older adult. On the one hand, being treated at a geriatric facility reduced his feelings of loneliness and helped him to understand that depression was common in later life. On the other hand, comparing himself to others on the ward deepened his sadness about ageing.

It’s also, in a way, disheartening to find yourself as part of this group that’s supposed to receive this help. We come there… if I may put it bluntly, we stumble in with our anxiety, and we recognize each other… we see each other, and it’s like… I can feel that it brings more sorrow. There’s a lot of sorrow surrounding it, very tangible sorrow, and there’s not much you can do about it either. (Lars)

### Obstacles cause powerlessness

This theme captures the powerlessness felt by participants when they could not access the care they needed. Participants described their efforts to navigate the healthcare system but often felt powerless when attempting to communicate their needs. Many reported feeling lost within the system as they waited for referrals, procedures or guidance on where to seek help. They described a gap between different healthcare units, where participants lacked someone to monitor their well-being or guide them to the appropriate assessments. Despite their efforts to seek care, participants had little ability to influence or control their access, leading to a sense of rejection during times of profound chaos and despair.

Martha faced a long wait in the queue for surgery for a progressive condition that made her housebound. She expressed feeling overlooked and uninformed as information regarding the queue time seemed arbitrary and was only provided on request. She said that the lack of communication and updates from healthcare professionals deepened her sense of powerlessness, which intensified her loneliness and despair. Reflecting on what kind of support she would have needed, Martha wished that a healthcare professional had reached out to her.

P:… if there had just been… yes, someone who understood what I was going through in some way.

I: that would have made a difference for you

P: Yes, the feeling would have been different, I wouldn’t have felt completely alone because I really did feel entirely alone in this and unable to do anything about it. (Martha)

In the months preceding Daniel’s suicidal act, he suffered from a severe depressive episode, felt out of sorts and tried numerous times to get a doctor’s appointment. However, due to the COVID-19 pandemic, access to healthcare was restricted, and he was prescribed antidepressants over the phone without any follow-up. The depression deteriorated, and Daniel expressed feeling rejected when he reached out for acute help.

It was at the (psychiatric) emergency room, you know, no, they weren’t good there. (…). They didn’t want to let anyone in. They didn’t want to let anyone in… unless they were half-dead or nearly dead. (Daniel)

In contrast to Daniel and Martha’s experiences of rejection, Lena exemplifies a more immediate despair driven by the lack of a healthcare response in an acute situation. Lena lived alone on the sixth floor of her apartment building. Lena struggled with pain after knee surgery, and at the time of her suicidal act, the pain was unbearable.

I tried to call for help using the alarm, but no one answered. (…) In the end, I stood in my bedroom window and screamed for help. “If I don't get help now, I'm going to jump”. (Lena)

### Healthcare that reduces agency

All participants sought care for their mental distress during the years leading up to their suicidal act. Several requested specific treatments based on their past experiences or preferences, but standardised treatment protocols limited their ability to influence their care in ways they found helpful. Others experienced uncertainties regarding their medication due to multiple prescribers and poor communication between medical units.

Two years prior to his suicidal act, Lars turned to primary care with a specific request to talk about his existential challenges, as he knew such psychotherapeutic interventions had been helpful when he was younger. He was informed that the available treatments in primary care were either pharmacological treatment, cognitive behavioural therapy (CBT) or a combination of both. Lars had little faith in either treatment but decided to try them. However, he discontinued both treatments early on, and his depression gradually worsened.

Yes, I spoke with a doctor at the primary care centre here, a younger doctor, a bit rushed and very… well, medically focused, as I perceived him. He didn’t seem to have any deeper interest in either psychiatry or existential questions. He referred me to CBT …… I tried it, but I didn’t find it helpful. (…) It became very checklist-oriented; she would mark boxes. And there was a strong emphasis from the therapist on not discussing underlying, deeper, or more abstract feelings. It was very much like, “we’re sticking to this schedule.” That’s how I experienced it. (Lars)

Several participants experienced that communication between various medical doctors and healthcare providers at different care levels led to confusion about their prescribed treatments. Kate described that her medication was adjusted at the inpatient ward after her suicidal act and paid close attention to adhere to the new medication regimen since she had bipolar disorder and was well aware of the importance of her medication for mood stabilisation. Kate experienced a setback after discharge, when her new psychiatrist at the outpatient clinic abruptly discontinued two of her medications.

I was so angry, so angry. Because he had stopped both my sleeping pill and my sedative, and I hadn’t slept for three nights when I finally got hold of him (…). (Kate)

### Restored hope through trust and validation

This theme highlights suicide preventative aspects of healthcare interactions that occurred primarily in the aftermath of the suicidal act. It encompasses three key aspects: feeling listened to in trusting treatment relationships; trust in care providers was restored, thanks to a holistic approach that addressed both medical and psychological needs; and attention to individual needs and recovery laid the foundation for self-care.

Participants described that when they had been able to talk about their experiences in validating and trusting treatment relationships, it eased their experience of burdensomeness and made them feel less alone in their struggles. After his suicidal act, Lars, whose CBT sessions in primary care prior to the suicidal act had not been helpful, started sessions with a psychologist at the geriatric psychiatric clinic.

I think the conversations with a psychologist have worked well; they have actually been helpful. They have helped me sort through a tangled thicket of thoughts and see things more clearly… (…) And it has worked because of the good connection I have with that particular therapist, which really relies heavily on trust. (Lars)

The second aspect of this theme encompasses how attendance to psychological and medical needs restored trust in care and increased participants’ engagement in their recovery. For the participants represented here, it was central that the doctors took a holistic approach, counterbalancing earlier experiences of feeling passed around between different medical units. For example, Martha, who had lost faith in the healthcare system before her suicidal act, expressed how her psychiatrist’s holistic approach restored her trust. She got an earlier slot for surgery, as well as help for several other somatic issues.

I didn’t feel like I had a good relationship with the primary care centre. (…) There wasn’t anyone I trusted, so I didn’t say anything. But then… when I saw a doctor [geriatric psychiatrist] here, I thought, since I have cataracts, they should be able to write a referral for me. And he said he would…… And then I also have tremors, I have essential tremor, and I had read that the best medication for it is something called propranolol. …. he prescribed it for me. I wasn’t sure if that would work, because, well, they’re the experts—you’re not really supposed to make suggestions as a patient. But it worked out, and he actually listened to me. (Martha)

Daniel also expressed that he regained trust in healthcare as the doctors at the geriatric psychiatric inpatient ward showed interest in his situation and encouraged him to talk about it, and this approach led to a referral for his medical needs as well.

I ended up in psychiatric care, and that was really good for me—it was fantastic. I felt like it was actually the best thing for me. There were good doctors, good staff, and good 'crazies' who were there with me… It was actually fun. And he [the geriatric psychiatrist] helped me too—I had a hernia here, an inguinal hernia….It was really big. So I asked him if he could help me with that …… He said, “I'll see what I can do.” Then they called me from the specialist hospital, and I got an appointment there. So, I went in and had surgery. He made it happen. (Daniel)

Some participants expressed that mental health professionals’ engagement in their needs and recovery opened up for continued self-care. Hugo appreciated that the doctors at the geriatric psychiatric inpatient ward listened when he expressed that staying in a single room made him unhappy. As his mental health improved after his need for social connection was met, he realised the significant impact loneliness had had on his mental health before his suicidal act.

I was allowed to change rooms and share a room with another fellow (…) we became friends… that’s when I started to feel better. (Hugo)

Later, Hugo actively participated in creating his personalised suicide prevention programme, which included regular visits to the senior activity centre.

Central to this theme is that when healthcare professionals actively listen and validate both medical and psychological needs, they help counteract hopelessness and suicidal feelings. Trust in healthcare professionals was essential for participants to openly discuss their needs, explore them further and take steps to address them after their suicidal act.

## Discussion

Findings highlight healthcare as an important arena for regulating the medical, psychological and social needs of older adults with suicidal issues. Most participants had serious physical health problems and were thus dependent on healthcare providers. Yet interactions with health professionals could result in amplification of age-related vulnerabilities, loss of agency and feelings of stigma. An overall interpretation can be summarised in the response of one participant who described her experience at a primary care consultation as “I felt like I was shrinking”. All described situations in which consultations with physicians increased vulnerabilities they already struggled with, including feelings of alienation, loneliness, worthlessness and, for some, suicidal ideation. Difficulties accessing care increased participants’ sense of powerlessness. Some of the participants were cognizant of their mental health needs but experienced obstacles that hindered them from managing their symptoms in a manner that had been helpful during previous illness episodes. Conversely, situations in which participants felt listened to in trustful and validating relationships helped restore self-respect and agency and ultimately increased hope for the future.

This study adds to the small, yet important, field of qualitative research on how older adults with self-harming behaviours experience their interactions with healthcare providers.^20^,^22^ By employing two interviews, as well as the IPA approach to analysis, this study yielded a multifaceted understanding of the perspectives of a group otherwise seldom represented in the literature on suicide prevention in clinical settings. The open-ended questions allowed participants to speak freely about the study topic, which enabled accounts free from the researcher’s prior assumptions. Since the analysis relied on retrospective accounts, narratives might have been impacted by recall, especially in persons with cognitive decline. The latter was evident in some participants with MoCA scores near the lower cut-off. Further, depressive symptoms during the suicidal process as well as at the time of interview likely affected participants’ rendition of events. This was alleviated somewhat through review of medical records to clear up issues regarding settings and timelines for various care contacts. Given that many patients declined to receive study information, purposive sampling was not an option. We do not know if those who declined participation might have had other types of experiences. However, we note that the included cohort is well-characterised and demonstrates heterogeneity regarding age group, sex, clinical diagnoses including alcohol use disorders, as well as depression scores, MoCA scores and physical illness burden.

Participants in our study reported that encounters with healthcare providers could either hinder or facilitate recovery, resonating with existing research on experiences of healthcare interactions in the aftermath of self-harm.^20^,^22^ Many participants reported feeling lonely, ignored or invalidated by healthcare professionals, reflecting what Bonnewyn *et al*^18^ describe as ‘existential loneliness’—a sense of disconnection and alienation. Experiences of loneliness and isolation are consistent with previous quantitative research, which identified perceived loneliness as a risk factor for suicidal attempts later in life.^31^ Consistent with Crocker *et al*^17^ who also used IPA, participants in our study described feeling like a burden to healthcare professionals and experiencing a lack of control over their treatment—both of which contributed to suicidal feelings. This aligns with the work of Gaily–Luoma *et al*^32^ who found that a lack of responsiveness and guidance in navigating recovery hindered progress among adults (of mixed ages) who received post-attempt care in Finland. It is therefore important, both in the prevention and post-care of suicidal acts, that healthcare interactions effectively address the most pressing subjective needs of service users. For many older adults, this includes recognising the role of ageing-related phenomena such as physical illness and functional limitations in the development of suicidal ideation.^33^ The primary phenomenon in healthcare interactions that facilitated recovery in our study was a restored hope through trust and validation, which fostered self-respect and agency. This finding aligns with Gaily–Luoma *et al*^32^ who emphasised that being regarded as worthy of help was crucial in fostering self-worth and empowering engagement in recovery. This resonates with Wand *et al*’s^20^ finding that feeling validated and listened to by healthcare professionals was stressed as important for recovery persons by persons aged 80 and above who took part in an interview in the aftermath of self-harm. Participants in our study appreciated the holistic approach in geropsychiatric services that addressed both medical and psychological needs, laying a foundation for self-care and increased agency. The central role of agency in recovering from suicidal feelings was given extra attention in Gaily–Luoma *et al*,^34^ based on interviews with Finnish adults of mixed ages who attended health and crisis services. Their results highlight the importance of the relational context provided by healthcare professionals in promoting goal-directed patient involvement in recovery.

Our results, taken together with those of others,^20^,^2233^ highlight the importance of adapting holistic care approaches. This may involve clinicians moving away from standardised treatment protocols and prioritising active listening and empathetic communication, ensuring patients feel heard and respected, for example, by acknowledging the patients’ narrative and validating their concerns. Poor communication between healthcare units and the lack of continuity of care diminished participants’ sense of agency, leaving them feeling frustrated and powerless, suggesting that the impact of poor communication between different healthcare units not only negatively affects clinicians’ ability to correctly assess and manage self-harm behaviours (as shown in Draper *et al*^35^ and Wand *et al*^20^) but may directly increase mental suffering in service users. Policymakers may incentivise the integration of mental healthcare within primary- and specialised-care settings to improve communication between healthcare providers and prevent fragmentation of care, for example, by implementing case managers in primary care. Some narratives highlighted the stigmatising nature of being perceived as an older adult requiring psychiatric care, which has been previously identified as a significant barrier to mental health service utilisation among older adults.^36^,^38^ To address this issue, and in line with suggestions for educational efforts targeting ageism within the healthcare system outlined before,^20^ suicide preventive workshops for healthcare personnel could be developed with a focus on how suicidality among older adults interacts with norms and attitudes within the healthcare system, such as ageism and stigmatising attitudes towards psychiatric patients. Involving older persons with lived experience^39^ to take part in the development of educational interventions and care development projects could provide valuable insights into areas in which provider behaviour may inadvertently harm patients. These efforts may help care professionals recognise and respond to the challenges facing older adults, including those associated with cognitive decline, functional disabilities and social isolation. Furthermore, enhancing medical training programmes to better prepare future healthcare providers to meet the needs of older adults is essential for improving the quality and accessibility of care.^20 33^

An important consideration is that our study was carried out in the context of geropsychiatric services in Sweden, and results may not be transferable to other settings. Further, the study was set up to inform the planning of an intervention for suicidal older adults with common mental disorders, which affects the transferability of findings to persons with other conditions, who would likely require longer-term treatment interventions.

Older adults’ experiences of healthcare need to be explored in other settings around the globe. Future research might focus on the development and testing of person-centred approaches to the care of older adults with mental and physical conditions. Early intervention studies are needed at the primary care level; behavioural health interventions need to be developed and tested for older adults with milder forms of depression, and these could include an existential component to help patients facing age-related challenges in daily life. Another knowledge gap in need of researcher attention is demonstrated by the dearth of intervention studies specifically designed to meet the needs of depressed and suicidal older adults in psychiatric services.

## Supplementary material

10.1136/bmjopen-2025-100636 online supplemental table 1

## Data Availability

No data are available.
