# Supplementary material for: Experiences of healthcare interactions before and after suicidal behaviour among older adults attending geropsychiatric services: an interpretative phenomenological analysis
Source: BMJ Open. 2025 May 30;15(5):e100636. doi: 10.1136/bmjopen-2025-100636 (PMC12128440; doi:10.1136/bmjopen-2025-100636)
Supplement: online supplemental table 1 [file bmjopen-15-5-s001.docx]

**Supplementary Table 1**. Example of coding scheme

| **Recording** | **Transcribed meaning unit** | **Phenomenological reading** | **Interpretative reading** | **Subtheme**  **(individual)** | **Theme**  **Group level** |
| --- | --- | --- | --- | --- | --- |
| **1** | “They didn’t give me my pills back…so I had two minor strokes”.  “I sat there by myself at the TV there and felt very sad. And they came by, but no one was paying any attention to me, no one said anything, there was this silence which didn’t make things better (…). Then I had one of those episodes where I got sad and stuff” | Malfunctioning health care leads to suffering  Sad when no one notices him crying | Trust in care has been decreased over a long period  Help-seeking leads to negative consequences.  Showing oneself vulnerable to health care professionals who do not react exposes loneliness and cause mental suffering | It’s lonely to be a patient  Malfunctioning health care increases mental suffering | Amplifying vulnerabilities |
| **2** | ”I wasn’t allowed to come…”  ”They discharged me”  ”They didn’t want to take anyone in”  “They don´t want to admit anyone … (you have to be) half-dead or almost dead” | Could not get a GP appointment although he felt that’s what he needed. Once again got a prescription by phone, no follow-up  Wished he had gotten to see a doctor sooner  Disappointed that he did not get to see a doctor in primary care when he called in asking for it | Health care is not available when one needs it  Feelings of being left out | Feeling powerless as a patient | Obstacles cause powerlessness |
| **3** | “I ended up in psychiatric care, and that was really good for me—it was fantastic. I felt like it was actually the best thing for me. There were good doctors, good staff, and good 'crazies' who were there with me… It was actually fun. And he [the geriatric psychiatrist] helped me too—I had a hernia here, an inguinal hernia….It was really big. So I asked him if he could help me with that …... He said, “I'll see what I can do.” Then they called me from the specialist hospital, and I got an appointment there. So, I went in and had surgery. He made it happen” | Helpful that the psychiatrist also made referrals for medical treatment.  Good people around.  Good to get help with what was causing problems.  The psychiatrist listens and tries to help.  Finally received help for the hernia he had sought treatment for several times before. | Contact with geriatric psychiatry made somatic care accessible. Receives holistic support.  At geriatric psychiatric ward, he becomes someone others are interested in – a whole person.  The care not only removed the pain but also gave something beyond that. Compassion, a holistic approach, and respectful treatment restored a sense of dignity — and with it, a renewed sense of hope. | Becomes visible during hospital stay on geriatric psychiatry ward. | Restored hope through trust and validation |
| **4** | “I left (the doctor’s office) and felt so sad. I was so sad. Because I didn’t ask for a miracle, I just asked for my medication”. | The new doctor does not give her the medication she needs  Feeling poorly treated by doctor  Does not want to go back to the primary care centre, but has no other choice | Help-seeking leads to negative consequences.  Trust in care has decreased over a long period  Lack of real contact with doctor | Feeling powerless as a patient  It’s lonely to be a patient | Amplifying vulnerabilities |
| **5** | “It got pitch black” | Her last hope vanishes when she is informed that she has to wait yet one more year for surgery | Critical point, her limit is passed. Increased despair and desperation leading to suicidal act  Help-seeking leads to negative consequences. | Feeling powerless as a patient | Obstacles cause powerlessness |
| **6** | “they sent me home” | Was not admitted to the psychiatric ward even though that’s what she wanted | Irresponsive psychiatric care  Needs unmet in psychiatric care. Is sent home against her will. Cannot take active part in her treatment | Feeling powerless as a patient | Obstacles cause powerlessness |
| **7** | “he told me that I was a real hypochondriac and they had taken lots of tests and he sent me home. And I got sad…..” | Gets really sad when doctor dismissed him as hypochondriac. | Help-seeking leads to negative consequences. | It’s lonely to be a patient  Malfunctioning health care increases mental suffering | Amplifying vulnerabilities |
| **8** | “That desperation started... I wasn't depressed then, but it was the knee that was the big problem, and I went crazy when I didn't get any response.” | Relates despair to acute pain, not to depression | Vulnerable position to depend on care that does not respond  Being dependent on others to access care is a vulnerable position | Feeling powerless as a patient | Obstacles cause powerlessness |
| **9** | “I spoke to some doctor, a young doctor, a bit rushed and very… medically oriented (…) with no interest in psychiatry or in existential matters. And I was referred to CBT (…) I gave it a try but didn’t find it helpful (…) the therapist really emphasized that we were not to talk about underlying or diffuse feelings.” | Contacted primary care and saw a young doctor who felt stressed and focused on medicines  Felt that the doctor was not interest in psychiatry or existential matters | Did not feel that he could affect the psychotherapeutic intervention, it was a standardized treatment protocol not matching his needs | Feeling powerless as a patient    Health care cannot meet the need to talk about existential challenges related to aging | Healthcare that reduces agency |
