# Supplementary material for: Experiences of healthcare interactions before and after suicidal behaviour among older adults attending geropsychiatric services: an interpretative phenomenological analysis
Source: BMJ Open. 2025 May 30;15(5):e100636. doi: 10.1136/bmjopen-2025-100636 (PMC12128440; doi:10.1136/bmjopen-2025-100636)
Supplement: Supplementary file 2 [file bmjopen-2025-100636.reviewer_comments.pdf]

PEER REVIEW HISTORY

BMJ Open publishes all reviews undertaken for accepted manuscripts. Reviewers are asked to complete a checklist review form and are provided with free text boxes to elaborate on their assessment. These free text comments are reproduced below.

ARTICLE DETAILS

Title (Provisional)

Experiences of health care interactions before and after suicidal behaviour among older adults attending geropsychiatric services: an interpretative phenomenological analysis

Authors

Sjöberg, Maja; Hed, Sara; Wiktorsson, Stefan; Berg, Anne Ingeborg; Strand, Jennifer; Doering, Sabrina; Waern, Margda

---

VERSION 1 - REVIEW

**Reviewer**	**1**
**Name**	**Gaily-Luoma, Selma**
**Affiliation**	**University of Jyväskylä, Psychology**
**Date**	**03-Mar-2025**
**COI**	**None**

Thank you for the opportunity to review this important and well executed study. Overall, the article is well written and accessible. The method is clearly described and consistent with the aims of the study. The findings are presented with adequate context and illustrative quotes. However, some concerns also arose and are detailed below.

INTRODUCTION

Page 5, Line 6: The authors state “Older adults have the highest rates of suicide worldwide”, but the WHO report referenced does not present suicide rates for older age groups. Please check the reference.

Page 5, Line 32-34: The authors write “Also, GPs may have difficulties identifying those at risk of attempting suicide because of the high proportion of physical complaints.” The meaning of this sentence is not clear (though I suspect the goal is to suggest that due to these complaints, the physician’s focus is drawn to physical issues and thus mental well-being and suicidality is not explored?). Please clarify.

METHOD

Page 7, Lines 23-35: The other exclusion criteria are clearly linked to the aims and method of the study (current or chronic condition that can be expected to severely affect the participants ability to give a coherent verbal account of past experiences) or relevant ethical concerns (current high risk). However, I am curious as to why the authors decided to exclude persons with a personality disorder diagnosis. Please comment.

DISCUSSION

Page 22, Lines 24-30: The qualitative method employed in this study is aimed at eliciting interpretations rather than "verifiable accounts" of events. I would thus consider rephrasing the limitations of the study in a way that is more consistent with the underlying epistemology; for example, there is no need to problematize depression-related or other "bias" in the participants' making of these interpretations. However, it is relevant to discuss how these findings may and may not be transferrable, i.e., how the selection of the participants may have highlighted or excluded certain perspectives relevant to a wider population of older adults with a history of suicidal acts.

Page 23, Lines 10-22: The authors write: "The older age of our participants (by approximately 8 years) compared to those in Crocker et al. suggests…"; but it is unclear to me how the qualitative findings would (or could) found these suggestions. Please clarify.

Page 23, Lines 23-47: The perceived importance (and inconsistency) of healthcare professionals hearing and addressing service users subjective needs (i.e., what they find the most important issues affecting their suicidal wish and quality of life) is a key finding in practically all studies concerning the experiences of suicidal individuals. I am thus not certain that the current findings justify the claim that these interactions are MORE relevant in older age groups. In my view, the current findings rather suggest that regardless of age, it is critically important (in both the prevention and post-care of suicidal acts) that healthcare interactions effectively address the most pressing subjective needs of service users, and that in older adults' case, it should be understood that these needs prominently include concerns with physical health and functioning.

Page 23, Lines 49-54: This is a misunderstanding concerning our study (Gaily-Luoma et al., 2024). A randomised clinical trial of the same population (i.e., suicide attempt survivors in the Helsinki Area receiving the Attempted Suicide Short Intervention Program) has been published (Arvilommi et al., 2022), but the samples of Arvilommi et al. (2022) and Gaily-Luoma et al. (2024) do not overlap. Instead, the findings of Gaily-Luoma et al. (2024) also concern service users' experiences in naturalistic treatment settings and emphasise that from the perspective of suicide attempt survivors, any and all healthcare interactions can be meaningful for either fostering or undermining recovery.

Arvilommi, P., Valkonen, J., Lindholm, L. H., Gaily-Luoma, S., Suominen, K., Ruishalme, O. M., Kukkonen, M., Sihvola, H., & Isometsä, E. (2022). A Randomized Clinical Trial of Attempted Suicide Short Intervention Program versus Crisis Counseling in Preventing Repeat Suicide

Attempts: A Two-Year Follow-Up Study. Psychotherapy and Psychosomatics, 91(3), 190–199. https://doi.org/10.1159/000521072

Gaily-Luoma, S., Valkonen, J., Holma, J., & Laitila, A. (2024). Suicide attempt survivors' recovery-related agency in the relational context of services: A qualitative analysis. BMC Public Health, 24(1), 3068. https://doi.org/10.1186/s12889-024-20459-z

---

Reviewer 2

Name Wand, Anne

Affiliation University of New South Wales

Date 04-Mar-2025

COI None

General comments

Thank you for the opportunity to review this interesting study exploring the experiences of older adults after a suicidal act in relation to interactions with healthcare providers. The study, which is part of a larger body of work is well designed and generally well written. The thematic analysis is thorough and emergent themes well-elaborated with illustrative quotations. The implications for healthcare providers and clinical practice are clear.

Although the same interviews with this group of older adult participants were also used for another paper (Hed et al 2024), the study aims in the present paper are quite specific and sufficiently different to warrant this second manuscript. Reading the Hed et al paper it became apparent that not all 'suicidal acts' in participants were actually behaviours with suicidal intent. This highlights the need for clear definitions of terms and to keep these terms used consistently throughout the paper. By current convention, 'suicidal behaviour without suicidal ideation' would be termed self-harm (see discussions in Edmondson et al 2016; Almeida et al 2012; Barrow et al 1997). It may mislead readers to use the word suicidal when the behaviour was not accompanied by the wish to die/end one's life. There is considerable literature highlighting that self-harm- with or without suicidal intent- is closely associated with suicide in older adults, so this does not change the importance of the paper.

The majority of qualitative literature used for comparison and contextualising the results is somewhat dated (over 10 years old) although there are more recent relevant qualitative studies (eg Van Orden et al 2015; Wand et al 2018; Draper et al 2018; Wand et al 2019; Conti et al 2020; Wand et al 2023). Findings from these other contemporary qualitative studies in the older adult context are largely consistent with the results of the present study (eg the theme of powerlessness and healthcare provider responses amplifying hopelessness, as well as previous descriptions of the inadvertent effect of healthcare provider responses sometimes exacerbating or amplifying the suicidal thoughts/original reasons for suicide; the

importance of validation etc). Inclusion of some of this recent prior work would strengthen this manuscript's findings.

Detailed comments

Abstract

Given questions about when the study aims for this study were determined in light of the broader study (see below comments- methods), it would be helpful to note in the Design or Participants section that this study re-examined interviews previously conducted for another study. The nomenclature issue should also be addressed.

The conclusion is missing a word in the first sentence (The).

Suggest adding the key-word self-harm and deleting those repeated in study title, eg attempted, to expand the likelihood of this paper being identified in a relevant literature search.

Introduction

See above re broader relevant literature which has not been included here or in the discussion.

Methods

At the start of the Methods it should be clearer that the interviews used for the study had already been conducted and an analysis previously published, but they are being re-examined in the present manuscript with a different aim/research question. This section currently reads like the study was conducted de novo. This section could be condensed with reference to the full methods in the original paper, then mentioning any specific new methods relevant to this specific manuscript.

It is unclear how the second interview for each participant contributed to data gathering. In the Hed et al paper the second interview is described as having "emphasis was placed on events related to the suicidal act and how the individual was coping," rather than following up on queries/themes emergent from the first interview pertinent to interactions with healthcare providers specifically, as the present manuscript implies. What was the original study aim? This is important to clarify in assessing whether the original research methods used and described here were designed to answer a different study question, and then the data (interviews) were repurposed to answer a different question, without the matched design to examine the healthcare provider aspect specifically. If this was the case, then the follow-up interview questions asked may not have been specific to the present study aim. When did these interviews take place in relation to the present study aim being developed?

Table 1 could include whether the self-harm was with suicidal intent. The labelling of the column 'months between index SA and interview', index SA needs to be relabelled index self-harm, as not all behavioural acts were suicide attempts.

Data collection- see points above regarding whether the follow-up questions were specific to this study or the parent/original study.

Data analysis- if this methodology is the same as was used in the Hed et al study, that paper could be referred to with the corresponding text on data analysis summarised more briefly here.

Although helpful to present an example of the coding scheme (Supplementary Table 1), it seems out of place to present this analysis (which should be new and specific to this study and therefore in the Results) in the Methods section. Perhaps it could be integrated into the Results, or the reader referred to a previous paper illustrating the IPA method.

Results

The theme, 'mirrored as a subject' seems to be about participants feeling validated and/or heard by healthcare providers (see the final sentence of this theme page 20). The phrasing of the theme title here does not seem to clearly capture this idea.

How did the SPIV educator's contribution (page 12) inform changes in interpretation of the analysis or elsewhere in the manuscript?

Discussion

New information is introduced about the medical records being used to verify accounts given by participants (page 21). This cross-checking is not noted in the Methods. How was this data from medical records used? Did it affect the analysis?

Please revise the wording regarding nomenclature for the 'suicidal act' throughout the discussion as per above comments.

Please incorporate more of the recent relevant qualitative studies in the discussion to better situate your results within the current literature. In addition, other authors have also recommended specific training for healthcare providers on suicide in older adults, particularly considering ageism and stigma; papers which are relevant and would support your argument (p23).

Limitations

-Why were people with personality disorder excluded? This cohort is also at greater risk of suicide and the sample includes people who self-harmed, not all with suicidal intent.

-Only participants with mild cognitive impairment were included. Why were people with more severe cognitive impairment excluded? Other qualitative work with older adults who have self-harmed has shown their ability to contribute meaningfully with in-depth interviews and it may have led to a more representative study sample, especially given the focus was people over 70 (where cognitive impairment is more prevalent).

Supplementary material (file 1)/Methods- Were there any specific questions asked in interview 2 or did these vary depending on the content of the individual's first interview?

How far apart were the 2 interviews for individual participants? The latter two pieces of data would be helpful to include in the Methods.

What is "Bilaga 2. Forskningplan"? It looks like the study protocol for the ethics application. Why is this included? Is it an Appendix or Supplementary material which therefore should be referred to in the main text?

VERSION 1 - AUTHOR RESPONSE

	**Reviewer #1** Selma Gaily-Luoma	**Authors' response:**
1	INTRODUCTION. Page 5, Line 6: The authors state "Older adults have the highest rates of suicide worldwide", but the WHO report referenced does not present suicide rates for older age groups. Please check the reference.	Thanks for catching this. We now cite the correct WHO source for suicide rates.
2	INTRODUCTION. Page 5, Line 32-34: The authors write "Also, GPs may have difficulties identifying those at risk of attempting suicide because of the high proportion of physical complaints." The meaning of this sentence is not clear (though I suspect the goal is to suggest that due to these complaints, the physician's focus is drawn to physical issues and thus mental well-being and suicidality is not explored?). Please clarify.	Yes, we meant that the physician's focus is drawn to physical issues and thus mental well-being and suicidality is not explored. Please see revised text on page 4, lines 77-80:<br><br>"Also, GPs may not recognize suicidal issues in their older patients because these individuals often present with physical symptoms. As a result, the physician's attention may be directed toward addressing these complaints, potentially overlooking underlying mental health concerns."
3	METHOD. Page 7, Lines 23-35: The other exclusion criteria are clearly linked to the aims and method of the study (current or chronic condition that can be expected to severely affect the participants ability to give a coherent verbal account of past experiences) or relevant ethical concerns (current high risk). However, I am curious as to why the authors decided to exclude persons with a personality disorder diagnosis. Please comment.	When we initiated the original study, we were in the very early stages of planning a brief psychosocial suicide preventive intervention for older adults with common mental disorders who presented at hospital in connection with a suicide attempt. We aimed to interview a sample similar to those who would present for such treatment. A short

		intervention would not be appropriate for most persons with personality disorders. This rationale is now included in the method section. Page 7, line 142-149.
4	DISCUSSION. Page 22, Lines 24-30: The qualitative method employed in this study is aimed at eliciting interpretations rather than “verifiable accounts” of events. I would thus consider rephrasing the limitations of the study in a way that is more consistent with the underlying epistemology; for example, there is no need to problematize depression-related or other “bias” in the participants’ making of these interpretations. However, it is relevant to discuss how these findings may and may not be transferrable, i.e., how the selection of the participants may have highlighted or excluded certain perspectives relevant to a wider population of older adults with a history of suicidal acts.	Thank you for this comment. We have deleted the word “bias” so the text now reads “Since the analysis relied on retrospective accounts, narratives might have been impacted by recall, especially in persons with cognitive decline.” (page 22, lines 468-469). Please see our new section on **transferability** at the end of our discussion, where this is addressed: “…..Further, the study was set up to inform the planning of an intervention for suicidal older adults with common mental disorders, which affects the transferability of findings to persons with other conditions who would likely require longer-term targeted treatment interventions.” (pages 24-25, lines 536-539)
5	Page 23, Lines 10-22: The authors write: “The older age of our participants (by approximately 8 years) compared to those in Crocker et al. suggests…”; but it is unclear to me how the qualitative findings would (or could) found these suggestions. Please clarify.	Thank you for pointing out this issue. We have revised this part of the discussion section, and this sentence has been removed.
6	DISCUSSION. Page 23, Lines 23-47: The perceived importance (and inconsistency) of healthcare professionals hearing and addressing service users subjective needs (i.e., what they find the most important issues affecting their suicidal wish and quality of life) is a key finding in practically all studies concerning the experiences of suicidal individuals. I am thus not certain that the current findings justify the claim that these interactions are MORE relevant in older age groups. In my view, the current findings rather suggest that regardless of age, it is critically important (in both the prevention and post-care of suicidal acts) that healthcare interactions effectively	Thank you for this important comment. We no longer make this claim. The sentence has been removed.

	address the most pressing subjective needs of service users, and that in older adults' case, it should be understood that these needs prominently include concerns with physical health and functioning.	
7	Page 23, Lines 49-54: This is a misunderstanding concerning our study (Gaily-Luoma et al., 2024). A randomised clinical trial of the same population (i.e., suicide attempt survivors in the Helsinki Area receiving the Attempted Suicide Short Intervention Program) has been published (Arvilommi et al., 2022), but the samples of Arvilommi et al. (2022) and Gaily-Luoma et al. (2024) do not overlap. Instead, the findings of Gaily-Luoma et al. (2024) also concern service users' experiences in naturalistic treatment settings and emphasise that from the perspective of suicide attempt survivors, any and all healthcare interactions can be meaningful for either fostering or undermining recovery.<br><br>Arvilommi, P., Valkonen, J., Lindholm, L. H., Gaily-Luoma, S., Suominen, K., Ruishalme, O. M., Kukkonen, M., Sihvola, H., & Isometsä, E. (2022). A Randomized Clinical Trial of Attempted Suicide Short Intervention Program versus Crisis Counseling in Preventing Repeat Suicide Attempts: A Two-Year Follow-Up Study. Psychotherapy and Psychosomatics, 91(3), 190–199. https://doi.org/10.1159/000521072<br><br>Gaily-Luoma, S., Valkonen, J., Holma, J., & Laitila, A. (2024). Suicide attempt survivors' recovery-related agency in the relational context of services: A qualitative analysis. BMC Public Health, 24(1), 3068. https://doi.org/10.1186/s12889-024-20459-z	We apologize for our confusion. We did not realize that ASSIP was being delivered within regular services for suicidal persons in Finland (this is not the case in Sweden) and simply assumed these were ASSIP RCT participants. The section has been rewritten, please see lines page 23, lines 504-505.
	**Reviewer #2** Dr. Anne Wand	**Authors' response**
1	Thank you for the opportunity to review this interesting study exploring the experiences of older adults after a suicidal act in relation to interactions with healthcare providers. The study, which is part of a larger body of work is well designed and generally well written. The thematic analysis is thorough and emergent themes well-elaborated with illustrative quotations. The implications for healthcare providers and clinical practice are clear.<br><br>1.1 Although the same interviews with this group of older adult participants were also used for another paper (Hed et al 2024), the study aims in the present paper are quite specific and sufficiently different to warrant this second manuscript. Reading the Hed et al	1.1 Nomenclature is important, and we would like to provide some details that relate to our choice to use the term "suicidal acts". The inclusion criteria for the qualitative interviews required that the act be classified as a suicide attempt in the patient's medical record, based on clinical assessment at the time. This is now made clear in the manuscript, page 6 lines 134-136 "Inclusion criteria were a

	paper it became apparent that not all 'suicidal acts' in participants were actually behaviours with suicidal intent. This highlights the need for clear definitions of terms and to keep these terms used consistently throughout the paper. By current convention, 'suicidal behaviour without suicidal ideation' would be termed self-harm (see discussions in Edmondson et al 2016; Almeida et al 2012; Barrow et al 1997). It may mislead readers to use the word suicidal when the behaviour was not accompanied by the wish to die/end one's life. There is considerable literature highlighting that self-harm- with or without suicidal intent- is closely associated with suicide in older adults, so this does not change the importance of the paper.<br>1.2.The majority of qualitative literature used for comparison and contextualising the results is somewhat dated (over 10 years old) although there are more recent relevant qualitative studies (eg Van Orden et al 2015; Wand et al 2018; Draper et al 2018; Wand et al 2019; Conti et al 2020; Wand et al 2023). Findings from these other contemporary qualitative studies in the older adult context are largely consistent with the results of the present study (eg the theme of powerlessness and healthcare provider responses amplifying hopelessness, as well as previous descriptions of the inadvertent effect of healthcare provider responses sometimes exacerbating or amplifying the suicidal thoughts/original reasons for suicide; the importance of validation etc). Inclusion of some of this recent prior work would strengthen this manuscript's findings.	suicide attempt registered in the patients' medical record". Although one of the findings from the first paper (Hed et al., 2024) was that participants described their suicidal acts as impulsive and, in many cases, difficult to make sense of, we believe it is important to distinguish this from suicidal intent.<br><br>Subjective accounts of intent, understanding, and impulsivity—shared months after the event—may differ significantly from the clinical evaluation conducted immediately following the act. For this reason, and for the sake of congruence with the nomenclature in the Hed et al publication we would like to retain the term "suicidal act" (SA). We added a footnote to Table 1 to explain that the episode was coded as a suicidal act by the assessing psychiatrist. We are of course willing to make the nomenclature change if it remains the wish of the reviewer.<br><br>1.2. Thank you for highlighting recent qualitative studies that examine the role of healthcare in relation to self-harm among older adults, from both their own perspectives and those of clinicians, carers, and community workers.<br><br>We apologize that these studies were missed in our initial search, likely due to limitations in our search strategy —specifically, omitting the term "self-harm." We appreciate your suggestions and have now

		incorporated key findings from Wand et al. (2018, 2019, and 2023) into the background section. These studies underscore the importance of relational dynamics between older adults who self-harm and healthcare professionals. Please see the revised section on page 5 lines 98-105, as well as the revised discussion where key findings from Wand et al (2018, 2019, 2023) and Draper et al (2018) and Conti et al (2020) have been included. Van Orden (2015) is now included in the Methods section, line 147-149.
2	ABSTRACT. Given questions about when the study aims for this study were determined in light of the broader study (see below comments- methods), it would be helpful to note in the Design or Participants section that this study re-examined interviews previously conducted for another study. The nomenclature issue should also be addressed.	We have added this information to the Design, now reading: "This study re-examined transcripts from a broader study involving experiences of older adults who took part in two separate semi-structured interviews focusing on their experiences before and after a suicidal act. Interpretative Phenomenological Analysis was applied." Please see our take on the nomenclature issue in response 1.1.
3	ABSTRACT. The conclusion is missing a word in the first sentence (The).	Thank you for pointing this out; the wording is fixed now.
4	ABSTRACT. Suggest adding the key-word self-harm and deleting those repeated in study title, eg attempted, to expand the likelihood of this paper being identified in a relevant literature search. .	We have now removed the keyword "attempted suicide" and added the keyword self-harm.
5	INTRODUCTION. See above re broader relevant literature which has not been included here or in the discussion.	Thank you. Relevant literature has been added in the introduction and discussion. Please see answer 1.2.
6	METHODS. At the start of the Methods it should be clearer that the interviews used for the study had already been conducted and an analysis previously published, but they are being re-examined in the present manuscript with a different aim/research question. This section currently reads like the study	Based on this comment, we realize that we have not clearly communicated how the present study differs from Hed et al. (2024). In the present study, we used the same

	was conducted de novo. This section could be condensed with reference to the full methods in the original paper, then mentioning any specific new methods relevant to this specific manuscript.	interview transcripts as Hed (2024), but the data analysis is new and was initially conducted by the first author (MS), who deliberately refrained from reviewing the data analysis from the Hed paper. We have now revised the first sentence of the method section, it now reads: "The current study is based on interview transcripts from a broader research project investigating older adults' subjective experiences of their suicidal process. Transcripts were re-examined and recoded, now with a specific focus on interactions with health care professionals." (page 6, line 116-119)
7	METHODS. It is unclear how the second interview for each participant contributed to data gathering. In the Hed et al paper the second interview is described as having "emphasis was placed on events related to the suicidal act and how the individual was coping," rather than following up on queries/themes emergent from the first interview pertinent to interactions with healthcare providers specifically, as the present manuscript implies. What was the original study aim? This is important to clarify in assessing whether the original research methods used and described here were designed to answer a different study question, and then the data (interviews) were repurposed to answer a different question, without the matched design to examine the healthcare provider aspect specifically. If this was the case, then the follow-up interview questions asked may not have been specific to the present study aim. When did these interviews take place in relation to the present study aim being developed?	Thank you for pointing out the need for clarity here. One of the aims of the broader research project was to capture personal experiences of help-seeking behaviour, treatment and health care. This study aim was determined prior to ethics application and thus prior to both interviews. The interview guide included questions about participants' interactions with health care providers. In other words, the broad study was designed to answer the study question posed in the current paper. Please see our revised description of the interview questions on, page 6, lines 121-125. This addition replaces supplementary file 1, which has been removed. We also now clarify in the revised text that participants spontaneously brought up their interactions with care professionals also in other parts of the interviews, not

		only in response to the interviewer's specific prompts. (page 6, lines 126-127)
8	METHODS. Table 1 could include whether the self-harm was with suicidal intent. The labelling of the column 'months between index SA and interview', index SA needs to be relabelled index self-harm, as not all behavioural acts were suicide attempts.	Given the nomenclature discussion (please see question 1.1) we have clarified that SA in Table 1 stands for Suicidal Act (not Suicidal Attempt).
9	METHODS. Data collection- see points above regarding whether the follow-up questions were specific to this study or the parent/original study.	Please see answer 7.
10	Data analysis- if this methodology is the same as was used in the Hed et al study, that paper could be referred to with the corresponding text on data analysis summarised more briefly here.<br>Although helpful to present an example of the coding scheme (Supplementary Table 1), it seems out of place to present this analysis (which should be new and specific to this study and therefore in the Results) in the Methods section. Perhaps it could be integrated into the Results, or the reader referred to a previous paper illustrating the IPA method.	As mentioned above we realize that we have not clearly communicated how the present study differs from Hed et al. (2024). As the present study used the same interview transcripts as Hed (2024), but as the data analysis is new, we believe it is necessary to include a detailed description of the data analysis, as it is unique to this study. We also hope that the coding scheme (Supplementary Table 1) still will be of interest. We have now clarified that the data analysis differs from that in Hed (2024) by adding the following sentences:<br>-" Transcripts were re-examined and recoded, now with a specific focus on interactions with health care professionals.." (lines 117-119)<br>-"For this study, transcripts were reanalysed and coded with the current research question in focus" (lines 193-194).
11	RESULTS. The theme, 'mirrored as a subject' seems to be about participants feeling validated and/or heard by healthcare providers (see the final sentence of this theme page 20). The phrasing of the theme title here does not seem to clearly capture this idea.	Thank you for this valuable input. We agree and after discussion within the author group the theme now reads: "Restored Hope Through Trust and Validation".
12	RESULTS. How did the SPIV educator's contribution (page 12) inform changes in interpretation of the analysis or elsewhere in the manuscript?	The SPIV educator did not take part in the actual analysis but commented that the findings

		were presented in a respectful manner, and that the content aligned with experiences of NGO staff. The educator's input helped us to define and refine implications of our findings (see added text on page 13, line 256), which catalysed a collaborative grant application to develop and test an educational intervention for physicians and nursing staff in somatic specialist care settings.
13	DISCUSSION. New information is introduced about the medical records being used to verify accounts given by participants (page 21). This cross-checking is not noted in the Methods. How was this data from medical records used? Did it affect the analysis?	There were some instances, especially in persons with cognitive impairment, where it was difficult to grasp the care context and timeline they were referring to. A check in the patient records cleared this up. This informed our interpretations in a few instances, but it did not affect the actual findings which emphasised the experiences of the participants. We have added the following to the Methods section:<br>Page 7, lines 158-160:<br>"The first author (MS) reviewed medical records **to complement participants' narratives with a verifiable account for information,** including psychiatric diagnoses (DSM-5), somatic diagnoses, psychiatric history, and health care contexts."
14	DISCUSSION. Please revise the wording regarding nomenclature for the 'suicidal act' throughout the discussion as per above comments.	Please see answer 1.1.
15	DISCUSSION. Please incorporate more of the recent relevant qualitative studies in the discussion to better situate your results within the current literature. In addition, other authors have also recommended specific training for healthcare providers on suicide in older adults, particularly considering ageism and stigma; papers which are relevant and would support your argument (p23).	The discussion and the references have been updated, please see answer to question 1.<br><br>We have also expanded this section with a review and meta-analysis (Burnes et al, 2019) showing that

		educational interventions can decrease ageism, especially when combined with intergenerational contact.
16	LIMITATIONS. Why were people with personality disorder excluded? This cohort is also at greater risk of suicide and the sample includes people who self-harmed, not all with suicidal intent.	The reason for exclusion of personality disorders is now stated in the methods section, page 7, lines 142-149: "The reason for excluding persons with personality disorders, psychotic illness or dementia was that the interview study was a first step in a larger initiative that would include testing of a brief intervention for suicidal older adults with common mental disorders. Suicidal persons with personality disorders, psychosis or dementia would most likely require more specific interventions. Further, we noted in our previous study of older adults hospitalized after a suicidal act[25] that cognitively impaired persons experienced some challenges when relating their personal experiences in the research interview."<br><br>Also, how this effects transferability is now included in the discussion lines 535-539.
17	LIMITATIONS. Only participants with mild cognitive impairment were included. Why were people with more severe cognitive impairment excluded? Other qualitative work with older adults who have self-harmed has shown their ability to contribute meaningfully with in-depth interviews and it may have led to a more representative study sample, especially given the focus was people over 70 (where cognitive impairment is more prevalent).	In line with the comment of Reviewer 1 above, we felt that having a clinical diagnosis of dementia could severely affect the participants' ability to give a coherent verbal account of past experiences, especially when these were not very recent. We did include persons with suspected early-stage dementia by including clinically non-demented persons with (relatively) low MoCa scores. We note that cognitive issues affected the narratives of some participants with MoCa scores close to the

		predetermined lower cut-off. While these persons did not fulfil criteria for a clinical dementia diagnosis, they experienced some challenges when relating previous experiences in varied care settings. Another concern was that a participant with dementia might have forgotten what was discussed during the first interview when they took part in the second interview 1-2 weeks later. We now address how our exclusion criteria may have influenced transferability in the discussion section, page 24-25, lines 535-539.
18.	Supplementary material (file 1)/Methods- Were there any specific questions asked in interview 2 or did these vary depending on the content of the individual's first interview? How far apart were the 2 interviews for individual participants? The latter two pieces of data would be helpful to include in the Methods.	The questions relevant to the current study were part of both Interview 1 and 2 and these can now be found in the Methods section (page 6, line 120-125). However, participants could spontaneously talk about interactions with health care at any point in the interview and that this material was also coded (line 125-127).<br><br>First interview: Did you reach out to anyone prior to the attempt, during or after? What was that like? Was there anything you found helpful? Was there anything you wish had been different?<br><br>Second interview: What type of treatment did you receive to help you manage/cope with suicidal thoughts/plans/actions? What has been helpful? What could have been different/more helpful<br><br>To enhance clarity, we have now deleted this as Supplementary material and

		included the questions in the Methods section (page 6).<br><br>The interviews were 1-2 weeks apart, please see page 6, line 130.
19	What is “Bilaga 2. Forskningplan”? It looks like the study protocol for the ethics application. Why is this included? Is it an Appendix or Supplementary material which therefore should be referred to in the main text?	Correct, it is the study protocol for the ethics application. We uploaded this file as extra material to adhere to the journal guidelines which request a study protocol. It should not be referred to in the main text, and the file has now been removed to avoid confusion.

VERSION 2 - REVIEW

Reviewer 1
Name Gaily-Luoma, Selma
Affiliation University of Jyväskylä, Psychology
Date 12-Apr-2025
COI

Thank you for your thorough addressing of my comments. I found all the revisions satisfactory and have no further concerns.

Reviewer 2
Name Wand, Anne
Affiliation University of New South Wales
Date 15-Apr-2025
COI

The authors have done an excellent job addressing both reviewers’ comments. The study methodology is now much clearer, distinguishing this piece of work from the broader study and overarching aims of their research program. I agree it is useful to include the example of the coding scheme in the paper, which can be retained in supplementary materials as per the authors.

Minor comments

Page 5 line 100 (paragraph 4 of introduction). The added discussion of our group's research work demonstrating the importance of the relational context between older people with self-harm and their healthcare providers is pertinent, but the expression of the findings of this work is not quite right. I'd suggest the following edit:

Wand et al.21,22 explicitly examined older adults' experiences of interactions with health care providers and found that the relational context with their health care professionals was relevant both before and after their self-harm.

Page 6 line 118 (Methods paragraph 1)- presumably the interviews had already been recorded for the original study. Hence suggest deleting "and recorded", which is confusing so that the sentence reads "Transcripts were re-examined for this substudy…."

Page 6 line 136 (Methods, paragraph 2 of participants and procedure)- It is now clearer that the inclusion criteria of a suicide attempt was determined by the assessing psychiatrist, not necessarily the patient participant's stated intent. I'd suggest adding one additional sentence to explain (as per the Hed paper which elaborates that not all suicidal acts were behaviours with suicidal intent from the older person's perspective) that this conclusion of the psychiatrist may have differed from the older person's explanation of the event at the time of the qualitative interview. This point is important in placing the participant's perspective at the centre of the analysis.

Page 21 (paragraph 2 of discussion) line 462 – please add more detail to the end of this sentence to clarify which role of healthcare older people experience, i.e. is it healthcare services or healthcare providers or healthcare interactions?

VERSION 2 - AUTHOR RESPONSE

Reviewer: 1

Selma Gaily-Luoma

Comments to the Author:

Thank you for your thorough addressing of my comments. I found all the revisions satisfactory and have no further concerns.

Reviewer: 2

Dr. Anne Wand, University of New South Wales

Comments to the Author:

The authors have done an excellent job addressing both reviewers' comments. The study methodology is now much clearer, distinguishing this piece of work from the broader study and overarching aims of their research program. I agree it is useful to include the example of the coding scheme in the paper, which can be retained in supplementary materials as per the authors.

Minor comments

Page 5 line 100 (paragraph 4 of introduction). The added discussion of our group's research work demonstrating the importance of the relational context between older people with self-harm and their healthcare providers is pertinent, but the expression of the findings of this work is not quite right. I'd suggest the following edit:

Wand et al.21,22 explicitly examined older adults' experiences of interactions with health care providers and found that the relational context with their health care professionals was relevant both before and after their self-harm.

Authors' response: We apologize for the confusion and thank the reviewer for the clarification. The sentence now reads (page 5, lines 98-101):

"Wand et al.[21 22] explicitly examined older adults' experiences of interactions with health care providers and found that **the relational context with their health care professionals was relevant both before and after their self-harm."**

Page 6 line 118 (Methods paragraph 1)- presumably the interviews had already been recorded for the original study. Hence suggest deleting "and recorded", which is confusing so that the sentence reads "Transcripts were re-examined for this substudy...."

Authors' response: The wording has now been adjusted, see page 6, line 118 "Transcripts were re-examined for this sub-study, now with a specific focus on interactions with health care professionals."

Page 6 line 136 (Methods, paragraph 2 of participants and procedure)- It is now clearer that the inclusion criteria of a suicide attempt was determined by the assessing psychiatrist, not necessarily the patient participant's stated intent. I'd suggest adding one additional sentence to explain (as per the Hed paper which elaborates that not all suicidal acts were behaviours with suicidal intent from the older person's perspective) that this conclusion of the psychiatrist may have differed from the older person's explanation of the event at the time of the qualitative interview. This point is important in placing the participant's perspective at the centre of the analysis.

Authors' response: We thank the reviewer for this suggestion and have now added the following sentence to the methods, page 6, lines 136-138:

"**It is important to note that for some participants, the psychiatrist's conclusion regarding suicidal intent differed from the older person's explanation of the act as described at the time of the qualitative research interview.[23]"**

Page 21 (paragraph 2 of discussion) line 462 – please add more detail to the end of this sentence to clarify which role of healthcare older people experience, i.e. is it healthcare services or healthcare providers or healthcare interactions?

Authors' response: This has now been clarified, see page 21, line 465:

“This study adds to the small, yet important field of qualitative research on how older adults with self-harming behaviours experience **their interactions with healthcare providers.**[20-22]”

VERSION 3 - REVIEW

**Reviewer**	**2**
**Name**	**Wand, Anne**
**Affiliation**	**University of New South Wales**
**Date**	**02-May-2025**
**COI**	

The manuscript is now suitable for publication.
